# Cortisol level and suicidal risk

**DOI:** 10.1186/s41983-018-0023-1

**Published:** 2018-08-24

**Authors:** Beuy Joob, Viroj Wiwanitkit

**Affiliations:** 1 Sanitaton 1 Medical Academic Center, Bangkok, 10330 Thailand; 2 Hainan Medical University, Haikou, China

Dear Editor,

The publication on “cortisol level and suicidal risk” by Ahmed et al. is very interesting (Ahmed et al. 2016). Ahmed et al. concluded that “There are relatively increased levels of morning and evening cortisol in major depressive disorder patients in comparison with controls; thoughts of death are positively associated with elevated morning and evening cortisol level (Ahmed et al. 2016)”. In fact, there are many factors that might results in aberration of cortisol level. In the present study, the control of confounding factor is questionable. The control of venipuncture pain and hemolysis which is an important primary inference on blood cortisol level should be considered (Lima-Oliveira et al. 2013; Snyder et al. 2004) and there is a remained question on the present study by Ahmed et al. (Ahmed et al. 2016) on the mentioned confounding factor. In clinical chemistry laboratory diagnostic test, the aberration of cortisol level can be seen in case with venipuncture pain and hemolytic samples (Lima-Oliveira et al. 2013; Snyder et al. 2004).
